# Effect of Enzymatic Lactose Hydrolysis on the Quality and Texture of Full-Fat Curd Cheese Produced Without Whey Separation

**DOI:** 10.3390/microorganisms13112471

**Published:** 2025-10-29

**Authors:** Małgorzata Ziarno, Dorota Zaręba, Iwona Ścibisz, Mariola Kozłowska

**Affiliations:** 1 Department of Food Technology and Assessment, Institute of Food Science, Warsaw University of Life Sciences—SGGW (WULS–SGGW), Nowoursynowska 159c St., 02-776 Warsaw, Poland; iwona_scibisz@sggw.edu.pl; 2 Professor E. Pijanowski Catering School Complex in Warsaw, 04-110 Warsaw, Poland; dorotazareba@gmail.com; 3 Department of Chemistry, Institute of Food Science, Warsaw University of Life Sciences—SGGW (WULS–SGGW), Nowoursynowska 159c St., 02-787 Warsaw, Poland; mariola_kozlowska@sggw.edu.pl

**Keywords:** curd cheese, enzymatic lactose hydrolysis, lactose-free dairy, texture properties, microbial stability, water-holding capacity

## Abstract

Lactic acid bacteria (LAB) play a crucial role in acid-curd cheese production by driving milk protein coagulation and forming metabolites that determine texture, safety, and flavor. This study investigated the effect of enzymatic lactose hydrolysis using β-D-galactosidase (Maxilact LX5000) on the quality of full-fat curd cheeses (16.5% and 20.8% dry matter) produced without whey separation. Cheeses were manufactured with or without prior lactose hydrolysis, inoculated with a mesophilic Flora Danica starter culture, and stored for 28 days at 4 °C. Chemical composition, sugar profile (HPLC), pH, LAB viability, textural properties (hardness, adhesiveness, and water-holding capacity), and sensory attributes were determined. Lactose hydrolysis completely eliminated lactose and increased glucose and galactose concentrations, without significant changes in protein, fat, or pH level. In our data, lactose was undetectable in hydrolyzed samples across storage, glucose/galactose exhibited only minor fluctuations, and LAB counts and pH remained stable, indicating a largely stable sugar profile and limited microbial activity under refrigeration. Hydrolyzed samples showed improved texture, especially higher hardness and moisture retention in low-dry-matter variants, while sensory characteristics were comparable to the control and free from excessive sweetness. These results demonstrate that enzymatic lactose hydrolysis is an effective tool for producing lactose-free curd cheese without compromising quality. This process can be recommended for sustainable whey-free cheese manufacture aimed at lactose-intolerant consumers.

## 1. Introduction

Curd cheese (also known as cottage cheese, quark, or twaróg) is a prominent product in the Polish dairy sector, with annual production exceeding 500,000 tons as of 2021 [1,2]. Twaróg, also referred to as tvorog in Russian or tvaroh in Czech and Slovak, is a traditional acid-set fresh cheese widely consumed across Central and Eastern Europe [3]. It is typically obtained by coagulating milk proteins through the action of lactic acid bacteria (LAB), rennet, or a combination of both [4,5,6,7]. Unlike rennet-coagulated cheeses, twaróg is valued for its delicate flavor, soft and crumbly texture, and high protein content, and it is often used in both savory and sweet dishes [8].

LAB play a dual role in curd cheese production: fermenting lactose and generating bioactive compounds such as organic acids and bacteriocins, which influence the product’s microbiological safety, flavor, and textural properties [4]. Despite its nutritional benefits, curd cheese may be unsuitable for individuals with lactose intolerance [9]. To address this, enzymatic hydrolysis using β-galactosidase (lactase) is employed to reduce the lactose content [10,11]. The origin of the enzyme significantly influences the hydrolysis outcome, depending on processing parameters [10]. Furthermore, lactose breakdown affects LAB activity, altering fermentation kinetics and metabolite composition [9,10]. Advances in immobilized enzyme systems also show promise for optimizing hydrolysis in industrial settings [12].

While the effects of lactose hydrolysis have been studied in various traditional and low-fat cheeses that involve whey separation [13,14,15], limited research exists on its application in zero-waste curd cheese systems, where all milk components, including whey proteins, are retained in the final matrix [16,17,18]. This approach not only enhances the nutritional value of the product but also eliminates the need for whey disposal, supporting sustainable production models [16,17,19,20].

Recent technological developments now allow for the manufacture of full-fat curd cheese without whey separation by recombining all milk constituents. These methods offer improved control over the protein-to-fat ratio, better nutrient retention, and facilitate microbiological management [17,21]. However, the implications of lactose hydrolysis in such systems, particularly on acidification profiles, bacterial metabolism, and textural attributes, remain underexplored due to the novelty and complexity of these processes [22,23,24].

In parallel, consumer trends show increasing interest in lactose-reduced dairy products that do not compromise on flavor, texture, or nutritional integrity [25,26,27]. Producing curd cheese without whey separation aligns with both sustainability goals and evolving consumer expectations, promoting efficient use of raw materials while maintaining high product quality [17,18].

The role of LAB is especially critical in this context. Strains such as *Streptococcus thermophilus* and *Lactobacillus* spp. not only contribute to product stability and shelf-life but also enhance nutrient bioavailability and support gut health [28,29]. Moreover, advances in fermentation technology now enable targeted modulation of metabolite synthesis, including key compounds like lactic acid and diacetyl, allowing for refined control over sensory properties while preserving probiotic functionalities [30,31]. Furthermore, emerging research explores natural additives to modulate flavor and improve textural properties in lactose-free curd cheese [32]. These innovations align with circular bioeconomy strategies and support the broader shift toward zero-waste dairy processing [17,33].

This study investigates the combined effect of enzymatic lactose hydrolysis and whey-free processing on the quality parameters of full-fat curd cheese. Specifically, it focuses on measurable indicators such as sugar profile, pH, and total lactic acid bacteria (LAB) counts during cold storage at 4 °C. To maintain consistency with the experimental scope, the discussion of LAB carbohydrate metabolism is limited to aspects that can be directly assessed under the present design. A broader metabolic context is provided through the cited literature [28,29,30,31,32,33,34]. Additionally, to situate this research within current production practices, Table 1 presents a comparative overview of three curd cheese manufacturing models, highlighting key differences in composition, residual lactose content, environmental impact, and target consumer profiles.

## 2. Materials and Methods

### 2.1. Preparation of Model Curd Cheeses Without Whey Separation and Experimental Design

Two variants of model curd cheese without whey separation were prepared under laboratory conditions: a full-fat cheese with 16.50% dry matter and a full-fat cheese with 20.80% dry matter. Low-pasteurized milk containing 2.0% fat and 3.3% protein (Mlekpol, Grajewo, Poland) served as the base material for the model cheese production. Low-pasteurized skimmed milk powder with 1% fat and 35% protein (Gostyń, Poland) and cream with 12% fat and 3.0% protein (Łowicz, Poland) were employed to adjust the dry matter content. The use of low-pasteurized skimmed milk powder and cream instead of fresh full-fat milk allowed for precise and reproducible adjustment of the dry matter, fat, and protein contents in the model cheese base. This approach ensured a controlled modification of the composition independent of natural variability present in raw full-fat milk and enabled standardized production of cheese variants with target dry matter levels.

Within each variant, two models of curd cheese were produced: one with standard lactose content in the raw material (samples LDM and HDM, respectively) and another with lactose-free raw material (lactose enzymatically hydrolyzed; samples LDM-LH and HDM-LH, respectively).

For each model cheese, 2 L of cheese premix was used. To this volume, 4 mL of β-D-galactosidase enzyme preparation (Maxilact LX5000, 5000 NLU/g, Danisco, Copenhagen, Denmark) was added to the lactose-free samples, followed by incubation at 6 °C for 24 h to hydrolyze lactose. Subsequently, all samples underwent pasteurization in a Stephan UMC 5 (Stephan Machinery GmbH, Hameln, Germany) at 90 °C for 15 s and were then cooled to an inoculation temperature of 28 °C. At this point, 0.05 g of freeze-dried mesophilic curd cheese starter (Flora Danica, Chr. Hansen, Hoersholm, Denmark) and 2 mL of previously prepared (diluted prior to addition to the cheese premix) aqueous rennet solution (Hannilase L 685, 685 IMCU/mL, Chr. Hansen, Hoersholm, Denmark) were incorporated into each sample. The Flora Danica starter culture from Chr. Hansen comprised the following mesophilic lactic bacteria: *Lactococcus lactis* subsp. *cremoris, Lactococcus lactis* subsp. *lactis*, *Lactococcus lactis* subsp. *diacetylactis*, and *Leuconostoc mesenteroides*. The samples obtained were transferred to sterile cylindrical glass containers (diameter: 7 cm) in 170 mL aliquots and incubated at 28 °C for 12 h (containers were tightly sealed to prevent dehydration during subsequent incubation and storage). Thereafter, they were cooled and stored at 4 °C for 28 days. All model curd cheese samples without whey separation were analyzed immediately upon collection and at 7-day intervals during refrigeration. Experiments were conducted in duplicate using different batches of milk, milk powder, and cream.

The following parameters of the model curd cheese samples were analyzed: dry matter, protein, fat, lactose, glucose, and galactose content; total count of starter culture bacteria; pH; selected texture characteristics (hardness, adhesiveness, and water-holding capacity); and sensory assessment. Direct measurement of lactic acid was not performed due to methodological limitations. Instead, pH monitoring was used as a proxy for acidification. However, this approach presents a notable limitation, as neither lactic acid nor titratable acidity was quantified. For clarity, pH is reported solely as an indirect indicator of acidification, and any interpretations regarding buffering capacity or acidification mechanisms should be regarded as tentative and hypothesis-generating rather than conclusive. Lactose, glucose, and galactose values below the HPLC detection threshold were reported as “not detected” (nd). All microbial activity assessments under refrigeration were based exclusively on these direct measurements. Dry matter was determined by oven-drying at 103 °C to constant mass (~4 h). Fat and protein contents were determined using the Van Gulik and Kjeldahl methods [35,36], respectively. All analyses were performed in triplicate.

### 2.2. Chemical Analysis

The sugar content (lactose, glucose, and galactose) was determined using high-performance liquid chromatography (HPLC) [37,38,39]. Sample preparation for sugar content analysis involved the following steps: homogenization of 10 g of sample in 10 g of deionized water using an Ultra-Turrax T10 homogenizer (IKA, Staufen, Germany) for 3 min and an ultrasonic bath (50B Tinget Poland, Starowa Góra, Poland) for 30 min, followed by centrifugation (16,000× *g*, 4 °C, 30 min) and filtration (0.22 µm PTFE filters, Merck Life Science Sp. z o.o., Poznań, Poland). Chromatographic analysis of the supernatants was performed using a DeltaChrom Pump Injector (S6020 Needle Injection Valve, Sykam, Fürstenfeldbruck, Germany), a DeltaChrom Temperature Control Unit (Sykam, 1 Fürstenfeldbruck, Germany), a Refractive Index Detector (S3580 RI Detector, Sykam, Fürstenfeldbruck, Germany), a pre-column Guard Column Sugar-D (10 mm × 4.6 mm, 5 μm; Cosmosil, Nacalai Tesque, Kyoto, Japan), and a column Sugar-D (250 mm × 4.6 mm, 5 μm; Cosmosil, Nacalai Tesque, Kyoto, Japan). The HPLC separation parameters were as follows: flow rate 1 mL/min, column temperature 30 °C, detector range RI 10,000 mV, and sample rate 2 Hz. The mobile phase consisted of a 75:25 (*v*/*v*) mixture of acetonitrile (HPLC grade, Sigma-Aldrich, Saint Louis, MO, USA) and deionized water. External standards of lactose, glucose, and galactose (HPLC-grade, POCH, Gliwice, Poland) were prepared at varying concentrations (0.005–0.25%) to generate calibration curves. Sugar concentrations are expressed as grams of sugar per 100 g of sample. All analyses were performed in triplicate.

### 2.3. Textural Analysis

The textural properties were evaluated using a penetration test. Textural analysis tests included the analysis of hardness and adhesiveness using a Brookfield CT3 Texture Analyzer (Brookfield Engineering Laboratories, Inc., Middleborough, MA, USA). Hardness was defined as the peak force required to achieve a given deformation during the compression cycle. Adhesiveness was defined as the work required to separate the probe from the sample surface, represented by the area of the negative peak. A single immersion of a TA4/1000 probe (Brookfield Engineering Laboratories, Inc., Middleborough, MA, USA) into the sample container (a filled cylinder with a diameter of 1 cm) was performed to a depth of 30 mm at a speed of 2 mm/s and a force of 98 N. The analysis was conducted at 6 °C. Hardness and adhesiveness values were calculated using TexturePro CT V1.4 Build 17 software (Brookfield Engineering Laboratories, Inc., Middleborough, MA, USA). The pH of the samples was measured directly with a pH meter (CP-505 Elmetron, Zabrze, Poland). The water-holding capacity (WHC) of the curd cheese samples was determined by centrifuging 40 g of sample at 16,128× *g* for 20 min at 4 °C, as described by Ziarno et al. [40]. The resulting liquid was removed and weighed. The WHC was calculated as: WHC = (m1/m2) × 100 (%), where m1 is the mass of the precipitate after centrifugation (g) and m2 is the mass of the sample (g). All analyses were performed in triplicate.

### 2.4. Microbiological Analyses

Total viable counts of starter culture bacteria in the curd cheese samples were determined using the plate count method. Twenty microliters of appropriately diluted sample was inoculated onto Petri dishes containing M17 agar (Merck, Darmstadt, Germany). Plates were incubated at 30 °C for 72 h under aerobic conditions. All analyses were performed in triplicate.

### 2.5. Sensory Analysis

Sensory evaluation of the curd cheese samples was conducted at 1 and 4 weeks of storage at 4 °C using quantitative descriptive analysis. A 5-point hedonic scale was employed to assess appearance, consistency, taste, smell, and overall acceptability for each sample (1 = extremely dislike; 5 = extremely like). A 23-member panel of potential consumers (12 female and 11 male students from the senior years of the Faculty of Food Sciences) participated in the evaluation. The panelists were presented with samples in 170 mL glass jars labeled with 3-digit codes. Prior to the evaluation, all panelists underwent specific training in descriptive sensory evaluation of dairy products and were familiar with the product category according to ISO 2023 standards [41]. Sensory assessments were performed simultaneously by each evaluator at room temperature (20 °C).

The study was carried out in accordance with the Declaration of Helsinki [42]. Respondents did not provide their names or contact information (including IP address) and could finish the survey at any stage, in accordance with the General Data Protection Regulation of the European Parliament (GDPR 679/2016) [43]. Considering the anonymous nature of the survey and the inability to track sensitive personal data of the respondents, this study did not require the consent of the ethics committee.

### 2.6. Statistical Analysis

Prior to analysis, data were tested for normality using the Shapiro–Wilk test. Homogeneity of variances was assessed using Levene’s test. Only data meeting the assumptions of the ANOVA were included in the parametric analyses. Analysis of variance (two-way ANOVA with interactions) was employed to identify differences among mean scores. Tukey’s honestly significant difference (HSD) test was subsequently conducted to pinpoint specific differences between means. Statistical significance was established at the *p* < 0.05 level. A linear mixed-effects model (LMM) was used to evaluate the influence of the fixed factors: “lactose hydrolysis” (yes vs. no), “dry matter content” (low: 16.50% vs. high: 20.80%), and “storage time” (0, 1, 2, 3, and 4 weeks). The interaction term between “lactose hydrolysis” and “dry matter content” was included in the model to assess combined effects. Storage time was treated as a repeated measure, with sample identity as a random effect to account for within-subjects correlations.

## 3. Results and Discussion

### 3.1. Detailed Chemical Analysis

These findings highlight the significant role of lactic acid bacteria and their metabolites, as they are directly responsible for lactose metabolism and the generation of bioactive compounds influencing both the nutritional and health-related properties of fermented dairy products. This study evaluated the initial chemical composition of model curd cheeses produced without whey separation, comparing low-dry-matter (LDM) and high-dry-matter (HDM) variants, as well as their lactose-hydrolyzed counterparts (LDM-LH and HDM-LH). These compositions are summarized in Table 2, detailing the dry matter, fat, and protein contents.

Compositional analyses were performed only at the initial time point (day 1) to assess the effects of formulation and lactose hydrolysis. While dry matter, fat, and protein are generally considered stable during short-term storage, we recognize that omitting their repeated measurement may limit mechanistic insight. Future longitudinal analyses are necessary to explore potential changes due to proteolysis, buffering interactions, and moisture migration. Therefore, although these parameters were assumed stable, their omission represents a limitation of the current sampling scheme, which focused instead on dynamic parameters like sugar content and pH.

To improve the clarity of data interpretation, all trends are described in relation to statistically significant comparisons and contextualized using compositional benchmarks across treatments.

In contrast to traditional tvorog-type curd cheeses [4,44], the present study adopted an intentionally modified process involving recombined cheese milk supplemented with milk powder and cream. These modifications altered the protein-to-fat ratio and dry matter content, diverging from conventional methods aimed at exceeding 25% dry matter or maintaining specific protein-to-fat ratios. Additionally, rennet was employed, unlike in tvorog production, which relies on acid coagulation, allowing for textural outcomes and nutrient retention through the no-whey-separation method. LAB activity during storage was closely monitored for its impact on sugar metabolism and acid production, with observable flavor shifts. However, without longitudinal compositional data, any connections to matrix buffering or proteolysis should be interpreted cautiously and are presented here as preliminary observations guiding future work.

This study demonstrated the effective use of β-D-galactosidase in producing lactose-free model curd cheeses. Across storage, lactose remained undetectable in LH samples, while glucose and galactose showed only modest changes, and both pH and total LAB counts remained essentially stable (Table 3 and Table 4). Accordingly, the data support a conservative interpretation: under 4 °C storage, the sugar profile is largely stable and microbial activity is limited, with no empirical basis here for asserting continuous glucose metabolism or preferential substrate utilization by LAB. Any mechanistic hypotheses are therefore beyond the resolution of the present measurements and are presented only as possibilities to be tested in future work.

The observed stability in pH despite lactose hydrolysis underscores the buffering interactions within the cheese matrix, but it also emphasizes the adaptive metabolic activity of LAB, which can modulate acid production depending on available substrates. An important limitation of this study is the lack of quantitative lactic acid measurement, which constrains the interpretation of pH stability. Furthermore, although lactose was hydrolyzed, residual galactose remained present. Future approaches should actively explore strategies to reduce galactose, e.g., by selecting *gal^+^* LAB strains or introducing enzymatic conversion steps.

A comprehensive literature review confirms that β-galactosidase converts lactose to glucose and galactose and that LAB display diverse pathways for hexose catabolism [34,45,46,47,48,49,50,51,52,53,54,55,56]. In our dataset, however, the observed monosaccharide trends during refrigeration were small in magnitude and coincided with stable pH and LAB counts, so we refrain from attributing these changes to specific pathway preferences. Future experiments that quantify organic acids (e.g., lactic acid), titratable acidity, and strain-level metabolism are required to rigorously evaluate such mechanisms.

While glucose is rapidly metabolized by LAB, galactose is often utilized more slowly [50,51,52,53], impacting fermentation dynamics. Recent genomic studies (e.g., on *Lactiplantibacillus plantarum*) highlight diverse sugar metabolism capabilities among LAB [54], reinforcing the need for careful strain selection. Optimizing galactose metabolism is essential for producing lactose- and galactose-free dairy products, especially for consumers with metabolic disorders. Selected *gal^+^* strains have shown promise in reducing galactose levels in fermented dairy [34,55,56], but strategies must also maintain sensory and nutritional quality. Overall, a deeper understanding of LAB sugar metabolism can guide the development of safer, tailored dairy products.

### 3.2. The Textural Properties

Textural parameters such as hardness, adhesiveness, and water-holding capacity (WHC) are key quality indicators in fresh cheese and are predominantly influenced by casein network architecture and protein–water interactions [57,58]. Our findings demonstrate that enzymatic lactose hydrolysis notably influenced these parameters, particularly in low-dry-matter (LDM) cheeses. Compared to control samples, the LDM-LH variants exhibited increased hardness, adhesiveness, and WHC across the storage period (Table 4), suggesting tighter protein matrix organization and improved moisture entrapment.

While direct microstructural or compositional analyses (e.g., SEM/CLSM imaging or proteolysis assays) were not conducted in this study, the observed trends align with previously reported correlations between network structure and mechanical properties in acid-rennet cheeses [57,59]. The mechanistic explanation proposed here, involving potential roles of glucose and galactose (products of lactose hydrolysis) in modulating intermolecular interactions and possibly acting as plasticizers or cross-linking agents [20,57,60,61], should be interpreted as hypothetical, based on literature-supported models. In contrast, the high-dry-matter (HDM) cheeses displayed only marginal changes in hardness, with a slight increase in adhesiveness observed in the HDM-LH samples. This contrast supports the assumption that in HDM matrices, the dense, pre-formed casein network restricts further cross-linking or water redistribution [57,62].

The more limited effect of hydrolysis in HDM cheeses can likely be attributed to restricted water mobility and a reduced capacity for structural reconfiguration due to lower porosity and stronger internal bonding [63]. The moderate increase in adhesiveness in the HDM-LH samples may reflect surface-level interactions between hydrolysis products and peripheral casein micelles, rather than significant core restructuring. The WHC remained high in both HDM variants, which is consistent with expectations for tightly cross-linked matrices with limited syneresis.

Although our study did not include direct measurements of proteolysis, compositional changes, or imaging, the proposed interpretations are consistent with prior reports that link protein cross-linking density to cheese hardness and adhesiveness [57,64,65]. Additionally, β-casein polymorphism and milk protein genotypes have been shown to affect micelle behavior and curd properties [66], offering relevant context for the observed differences.

Future work should incorporate microstructural imaging, rheological characterization, and analysis of proteolytic activity over storage to confirm these hypotheses and elucidate the specific molecular mechanisms involved. Moreover, compositional monitoring over time would aid in distinguishing between texture changes arising from water redistribution versus biochemical modifications. Exploring β-galactosidase concentration and hydrolysis duration may further enable texture customization in low-solid cheese systems, where the functional impact appears to be most pronounced. These findings support the dual role of lactose hydrolysis: as a nutritional intervention and as a functional strategy for modulating cheese texture, particularly in LDM formulations.

### 3.3. pH Analysis

The pH of curd cheeses is a key parameter influencing microbial stability, texture, and shelf life, and it is generally governed by the balance between acid production and the buffering capacity of the matrix [57,67]. Interestingly, in the present study, no significant differences in pH values were observed between hydrolyzed and non-hydrolyzed variants across both dry matter levels during 28 days of storage (Table 4). In the LDM group, pH ranged from 4.31 to 4.43 in both the control and hydrolyzed samples (LDM and LDM-LH), while in the HDM group, values remained between 4.36 and 4.47. Consistent with refrigerated storage and limited microbial activity, pH fluctuations were minimal across all treatments, supporting an interpretation of matrix-buffered stability. These minor variations occurred regardless of lactose hydrolysis, suggesting the presence of a robust intrinsic buffering system within the cheese matrix.

This observation contradicts the expectation that increased glucose availability in lactose-hydrolyzed samples would lead to enhanced lactic acid production by LAB, resulting in a sharper pH decline. A plausible explanation is that the buffering capacity of the curd components, primarily caseins, soluble proteins, and residual calcium phosphate, effectively neutralized the produced acid, thereby maintaining pH stability [67]. Additionally, the presence of whey proteins (not removed due to the no-whey-separation model) may have contributed to buffering via their amino acid side chains, which can act as acid–base reservoirs.

Although pH is commonly used as a proxy for acidification, relying solely on this parameter is a significant limitation. The absence of direct lactic acid quantification in this study limits the interpretation of acid production and acid–base equilibrium. Moreover, titratable acidity (TTA), a standard and accessible method to assess total acidity, was not performed, which further restricts the conclusions regarding the nature of the acidification process. Without either TTA or direct lactic acid measurements, it is impossible to definitively determine whether the observed pH constancy reflects low acidogenesis or a strong buffering effect by the matrix [57,67].

Similarly, proteolytic activity during storage, which could modify the protein profile and thus influence buffering capacity, was not assessed, as changes in protein content or peptide release were not monitored. Consequently, all claims linking pH stability to buffering or LAB metabolism remain speculative and should be interpreted with caution.

Future studies should incorporate both direct measurement of lactic acid concentrations and titratable acidity, as well as analysis of proteolytic changes over time, in order to more accurately determine the respective contributions of microbial metabolism, matrix composition, and biochemical transformations to pH behavior [57,67]. Furthermore, the samples with a higher dry matter content (HDM and HDM-LH) demonstrated slightly greater pH stability, consistent with earlier findings that increased protein concentration enhances the system’s buffering capacity [57,67]. This is particularly important from a technological perspective, as controlling pH is essential for maintaining curd integrity and inhibiting undesirable microbial growth during storage. Overall, the findings underscore that matrix composition, not only LAB metabolism, plays a critical role in determining pH behavior in lactose-free or low-lactose cheese systems.

### 3.4. Total Viable Counts of Starter Culture Bacteria

The influence of enzymatic lactose hydrolysis on the growth and viability of lactic acid bacteria (LAB) in cheese is a critical factor affecting product quality, particularly its sensory and textural attributes. In our study, microbiological analysis based on LAB population counts (expressed as log CFU/g) revealed no statistically significant effect (*p* > 0.05) of enzymatic lactose hydrolysis on LAB viability during 28 days of refrigerated storage (Table 4). Initial counts ranged from 8.5 to 8.7 log CFU/g on day 0 and declined slightly to 8.0–8.1 log CFU/g by week 4 across all cheese formulations (LDM, HDM, LDM-LH, and HDM-LH), regardless of dry matter content or hydrolyzed lactose presence. Together with a stable pH, these data suggest bacterial survival rather than active proliferation, consistent with the reduced metabolic activity expected at 4 °C in fresh, whey-retaining curd matrices.

Although enzymatic hydrolysis increased the availability of glucose and galactose, theoretically more accessible carbon sources than intact lactose, this did not result in increased LAB proliferation or acidification, as pH values remained stable and no significant changes in LAB counts were observed. This finding challenges the initial assumption that hydrolysis would stimulate LAB metabolic stability over storage, particularly in *Lactococcus lactis* subsp. *lactis*, a known lac^−^/cit^−^ strain incapable of metabolizing lactose or citrate [68]. The Flora Danica starter culture used contains *Lactococcus lactis* subsp. *cremoris, L. lactis* subsp. *lactis*, L. *lactis* subsp. *diacetylactis*, and *Leuconostoc mesenteroides*, which together form a mesophilic LD-type culture widely used in fresh curd cheese and buttermilk production [4,69]. These strains are typically associated with metabolic activities influencing flavor; however, we did not directly assess acid production, enzymatic activity, or transcriptomic indicators of metabolic shifts, which limits our ability to draw firm conclusions regarding LAB-driven modulation of the cheese matrix.

While monosaccharides were available in the LH samples, the lack of a proliferation boost suggests the presence of buffering or regulatory mechanisms within the matrix (such as pH homeostasis, limited substrate diffusion, or interspecies microbial competition) that may restrict metabolic responses. It is also plausible that genetic constraints or repression of specific catabolic pathways prevented efficient utilization of glucose or galactose by some strains. Moreover, the dry matter content (LDM vs. HDM) significantly affected textural properties such as hardness and adhesiveness but did not influence LAB viability, further indicating that LAB compositional stability is robust to both matrix density and enzymatic modulation of lactose content.

Importantly, our analysis reported only total LAB counts, without distinguishing between individual strains or assessing strain-specific responses. Given known differences in sugar metabolism and flavor compound production, it is possible that compositional or functional shifts occurred within the LAB community that were undetected by the total count methodology. Future studies incorporating strain-level quantification, molecular profiling, TTA, enzymatic assays, and metabolic flux analysis are essential to fully elucidate the biochemical consequences of lactose hydrolysis on microbial metabolism and cheese maturation processes.

### 3.5. Sensory Evaluation

Our research focused on the influence of enzymatic hydrolysis of lactose on the sensory characteristics of model curd cheeses without whey separation (Table 5). The objective was to determine whether the reduction in lactose content would affect the perceived sweetness of the cheese, as expected and in accordance with the results of earlier studies by Mendoza et al. [49], who demonstrated a correlation between the intensity of lactose hydrolysis and excessive sweetness in dairy products. The results of the sensory analysis showed that the HDM and HDM-LH samples received the highest scores for flavor and overall acceptability in both the first and fourth week of storage. This suggests that the higher dry matter content may have had a greater impact on sensory perception than the lactose content itself. However, it is worth noting that for most sensory parameters, no statistically significant differences were observed between the hydrolyzed and control samples (*p* > 0.05), which confirms the sensory stability of the products regardless of lactose hydrolysis.

For a more detailed analysis of the complex interactions between factors, a two-way analysis of variance (ANOVA) was performed, including the interaction between sample type (LDM, LDM-LH, HDM, and HDM-LH) and storage time (1 vs. 4 weeks). The results indicated a significant interaction between these factors concerning flavor scores (*p* < 0.05), pointing to a complex influence of both composition and storage time on the perception of sweetness and other flavor attributes. This refines the earlier conclusions; while enzymatic lactose hydrolysis did not change the perception of sweetness to a statistically significant degree in the one-factor analysis, the significant interaction effects suggest the need for further sensory analysis over longer storage periods or at higher degrees of hydrolysis.

The lack of a clearly increased perception of sweetness in samples with hydrolyzed lactose may also be attributed to possible metabolic transformations of the hydrolysis products (glucose and galactose) during refrigerated storage. These products might have been partially utilized by the cheese microflora, thereby reducing their impact on the sensory profile. In this context, it is worth highlighting that although only total lactic acid bacteria counts were reported, the starter culture used contained several strains with potentially varied metabolic properties, particularly regarding the utilization of glucose and galactose. Strain differences, especially in the ability to ferment individual monosaccharides, may affect the flavor and textural properties of the cheese, which were not fully accounted for in this analysis. Future research should include the identification and analysis of the activity of individual bacterial strains to better understand their role in shaping the organoleptic characteristics of dairy products. The stability of sensory perception may be related to various factors such as hydrolysis conditions, masking of sweetness by other flavor compounds formed during cheese ripening, or the sensory thresholds of the tasting panel. The results obtained are important for producing lactose-free or reduced-lactose dairy products. It is possible to adjust the lactose content in cheeses without negatively affecting the taste, which may interest producers seeking new product variants. Further research should focus on the balance between enzymatic lactose hydrolysis and sensory attributes, examining the impact of different hydrolysis conditions or enzyme concentrations on sweetness and other key flavor components. Additionally, a better understanding of consumer preferences and sensory thresholds can provide additional information on the optimal formula for dairy products with hydrolyzed lactose. In addition, future studies should analyze the profile of aroma compounds using GC-MS to understand whether lactose hydrolysis influences flavor profile or sweetness masking.

In summary, our research indicates that lactose reduction via enzymatic hydrolysis is a sensory-safe technology for curd cheeses without whey separation. These findings may serve as a starting point for the development of novel dairy products targeted toward consumers with lactose intolerance, without compromising sensory quality. It is advisable to expand the sensory panel size and conduct targeted consumer preference tests on specific attributes such as sweetness, acidity, and buttery notes.

## 4. Conclusions

Enzymatic lactose hydrolysis proved to be an effective strategy for producing full-fat curd cheese without whey separation, completely eliminating residual lactose while maintaining desirable physicochemical, microbiological, and sensory qualities. Under refrigerated storage, the sugar profile (glucose/galactose) exhibited only minor changes and both pH and total LAB counts remained stable, supporting the view of limited microbial activity at 4 °C in this system. Compared to traditional acid-curd and whey-separated systems [13,14,22], the applied approach ensured higher nutrient retention, reduced environmental impact, and preserved lactic acid bacteria viability during storage. Accordingly, mechanistic assertions about preferential substrate utilization were removed in favor of data-driven statements. Future work should explicitly couple sugar measurements with organic-acid quantification, titratable acidity, and strain-resolved analyses to test mechanistic hypotheses.

Despite the full conversion of lactose to glucose and galactose, no significant changes in pH or LAB populations were detected, consistent with findings from Guinee et al. [22] and Lepesioti et al. [24], who also noted buffering effects of casein-rich matrices. The observed improvements in hardness, adhesiveness, and water-holding capacity, particularly in the low-dry-matter variants, correspond with previous reports linking hydrolyzed carbohydrate profiles with enhanced protein-water interactions in fresh cheeses [57,60,62].

In sensory terms, the absence of excessive sweetness or texture degradation aligns with studies by Mendoza et al. [49] and Airouyuwa et al. [32], confirming that lactose hydrolysis can be applied without compromising consumer acceptability. Therefore, the combination of enzymatic lactose hydrolysis and whey-free processing represents a promising, sustainable approach for developing lactose-free dairy products comparable or superior to conventional methods in terms of both quality and functionality.

Future work should address residual galactose reduction through the use of galactose-positive LAB strains [55,56] or additional enzymatic steps, and quantify organic acid production to better understand buffering mechanisms [30,67]. Scaling the process to pilot or industrial conditions, with targeted rheological and consumer analyses, will further validate its technological and commercial potential.

## Figures and Tables

**Table 1:** Comparison of traditional vs. whey-free curd cheese production models (based on [9,10,13,14,16,17,19,20,22]).

Feature	Traditional (Whey-Separated)	Whey-Free (Lactose-Containing)	Whey-Free + Lactose-Hydrolyzed (This Study)
Lactose content	high	moderate	low
Galactose/glucose content	low	low	high (from hydrolysis)
Nutrient retention	partial	high	high
Environmental footprint	higher (whey disposal)	lower	lowest
Microbial activity (lactic acid bacteria)	controlled by lactose input	sustained by residual lactose	modulated by glucose/galactose availability
Target consumers	general, lactose-tolerant	general	lactose-intolerant + health-conscious
Production complexity	standard	moderate	higher (requires enzyme control)

**Table 2:** Composition of model curd cheeses (without whey separation) with low and high dry matters, with or without lactose hydrolysis (mean and SD).

Sample Code	Dry Matter Content (% *w*/*w*)	Fat Content (% *w*/*w*)	Protein Content (% *w*/*w*)
LDM	16.5 ± 0.4 ^a^ ^1^	10.86 ± 0.3 ^a^	4.99 ± 0.2 ^a^
LDM-LH	16.5 ± 0.4 ^a^	10.86 ± 0.3 ^a^	4.99 ± 0.2 ^a^
HDM	20.8 ± 0.4 ^b^	11.03 ± 0.2 ^b^	3.74 ± 0.3 ^b^
HDM-LH	20.8 ± 0.4 ^b^	11.03 ± 0.2 ^b^	3.74 ± 0.3 ^b^

^1^ Explanatory notes: Different superscripts within the columns for the results of a specific parameter show a significant difference at *p* < 0.05. Legend: LDM, curd cheese samples with 16.50% of dry matter and the standard lactose content; LDM-LH, lactose-free curd cheese samples with 16.50% of dry matter; HDM, curd cheese samples with 20.80% of dry matter and the standard lactose content; HDM-LH, lactose-free curd cheese samples with 20.80% of dry matter.

**Table 3:** Sugar content in full-fat curd cheese without whey separation stored refrigerated for 28 days (mean and SD).

Storage Time (Week)	0	1	2	3	4
Sample Code					
Lactose content (%)					
LDM	4.25 ^a 1^ ± 0.24	2.50 ^b^ ± 0.08	2.23 ^b^ ± 0.21	2.10 ^b^ ± 0.21	1.96 ^b,c^ ± 0.20
LDM-LH	nd ^d^	nd ^d^	nd ^d^	nd ^d^	nd ^d^
HDM	4.35 ^a^ ± 0.10	2.25 ^b^ ± 0.17	2.00 ^b,c^ ± 0.09	1.87 ^c^ ± 0.10	1.64 ^c^ ± 0.11
HDM-LH	nd ^d^	nd ^d^	nd ^d^	nd ^d^	nd ^d^
Glucose content (%)					
LDM	nd ^a^	0.15 ^a,b^ ± 0.06	nd ^a^	nd ^a^	0.05 ^a,b^ ± 0.00
LDM-LH	1.70 ^c^ ± 0.14	1.45 ^b^ ± 0.10	1.38 ^b^ ± 0.10	1.32 ^b^ ± 0.11	1.22 ^b^ ± 0.20
HDM	nd ^a^	0.10 ^a^ ± 0.00	0.01 ^a^ ± 0.00	nd ^a^	nd ^a^
HDM-LH	1.93 ^d^ ± 0.10	1.65 ^c^ ± 0.17	1.60 ^c^ ± 0.08	1.54 ^b,c^ ± 0.11	1.40 ^b^ ± 0.04
Galactose content (%)					
LDM	nd ^a^	1.63 ^b^ ± 0.10	1.75 ^b^ ± 0.10	1.78 ^b,c^ ± 0.08	1.70 ^b^ ± 0.10
LDM-LH	2.48 ^d^ ± 0.10	2.40 ^d^ ± 0.10	2.44 ^d^ ± 0.13	2.34 ^d^ ± 0.08	2.31 ^d^ ± 0.11
HDM	nd ^a^	1.83 ^b,c^ ± 0.10	1.70 ^b^ ± 0.00	1.75 ^b^ ± 0.00	1.66 ^b^ ± 0.00
HDM-LH	2.75 ^e^ ± 0.21	2.68 ^e^ ± 0.24	2.69 ^e^ ± 0.18	2.74 ^e^ ± 0.08	2.70 ^e^ ± 0.10

^1^ Explanatory notes: Statistical analysis: A linear mixed-effects model (LMM) was used to assess the effects of: Lactose hydrolysis (yes vs. no), Dry matter content (low: 16.50% vs. high: 20.80%), Storage time (0 to 4 weeks). The model included an interaction term (lactose hydrolysis × dry matter content). Storage time was treated as a repeated measure, with sample ID as a random effect to account for within-subject correlations. Superscript letters indicate statistically significant differences within rows (*p* < 0.05). nd—not detected; sugar concentration was below the detection limit of the analytical method. Sample codes: LDM—low dry matter (16.50%), non-hydrolyzed (with lactose), LDM-LH—low dry matter, lactose-hydrolyzed, HDM—high dry matter (20.80%), non-hydrolyzed (with lactose), HDM-LH—high dry matter, lactose-hydrolyzed.

**Table 4:** Quality parameters of full-fat curd cheese without whey separation during 28-day refrigerated storage (mean and SD).

Storage Time (Week)	0	1	2	3	4
Sample Code					
Hardness (N)					
LDM	3.2 ^a^ ± 0.2	3.1 ^a^ ± 0.2	3.5 ^a^ ± 0.2	3.9 ^a,b^ ± 0.2	3.1 ^a^ ± 0.3
LDM-LH	4.9 ^b^ ± 0.5	4.9 ^b^ ± 0.1	5.2 ^b^ ± 0.2	5.0 ^b^ ± 0.2	4.8 ^b^ ± 0.1
HDM	9.1 ^c^ ± 0.4	9.1 ^c^ ± 0.8	8.8 ^c^ ± 0.4	9.0 ^c^ ± 0.5	9.0 ^c^ ± 0.4
HDM-LH	8.7 ^c^ ± 0.5	9.2 ^c^ ± 0.7	9.2 ^c^ ± 0.5	9.1 ^c^ ± 0.4	9.0 ^c^ ± 0.4
Adhesiveness (N)					
LDM	0.8 ^a^ ± 0.1	0.8 ^a^ ± 0.1	0.8 ^a^ ± 0.1	0.8 ^a^ ± 0.1	0.8 ^a^ ± 0.1
LDM-LH	2.3 ^b^ ± 0.8	2.3 ^b^ ± 0.3	2.5 ^b^ ± 0.3	2.3 ^b^ ± 0.2	2.4 ^b^ ± 0.2
HDM	3.9 ^c^ ± 0.6	4.0 ^c^ ± 0.4	3.9 ^c^ ± 0.5	3.9 ^c^ ± 0.4	4.1 ^c^ ± 0.4
HDM-LH	5.0 ^d^ ± 0.3	4.7 ^d^ ± 0.2	4.7 ^c,d^ ± 0.7	4.7 ^c,d^ ± 0.5	4.8 ^d^ ± 0.5
Water-holding capacity (%)					
LDM	82 ^a^ ± 4	83 ^a^ ± 4	84 ^a^ ± 4	82 ^a^ ± 4	84 ^a^ ± 4
LDM-LH	98 ^b^ ± 5	95 ^b^ ± 5	95 ^b^ ± 5	96 ^b^ ± 5	97 ^b^ ± 5
HDM	100 ^b^ ± 5	100 ^b^ ± 5	100 ^b^ ± 5	97 ^b^ ± 5	100 ^b^ ± 5
HDM-LH	100 ^b^ ± 5	99 ^b^ ± 5	100 ^b^ ± 5	99 ^b^ ± 5	100 ^b^ ± 5
pH					
LDM	4.43 ^a^ ± 0.18	4.42 ^a^ ± 0.13	4.40 ^a^ ± 0.21	4.34 ^a^ ± 0.14	4.31 ^a^ ± 0.22
LDM-LH	4.43 ^a^ ± 0.17	4.43 ^a^ ± 0.20	4.37 ^a^ ± 0.22	4.36 ^a^ ± 0.16	4.33 ^a^ ± 0.13
HDM	4.47 ^a^ ± 0.22	4.47 ^a^ ± 0.20	4.45 ^a^ ± 0.22	4.44 ^a^ ± 0.22	4.42 ^a^ ± 0.16
HDM-LH	4.42 ^a^ ± 0.10	4.42 ^a^ ± 0.22	4.37 ^a^ ± 0.19	4.37 ^a^ ± 0.17	4.36 ^a^ ± 0.22
Population of lactic acid bacteria (log(CFU/g))					
LDM	8.6 ^a^ ± 0.1	8.4 ^a^ ± 0.3	8.4 ^a^ ± 0.2	8.3 ^a^ ± 0.2	8.1 ^a^ ± 0.2
LDM-LH	8.5 ^a^ ± 0.3	8.3 ^a^ ± 0.3	8.2 ^a^ ± 0.2	8.1 ^a^ ± 0.2	8.0 ^a^ ± 0.2
HDM	8.5 ^a^ ± 0.4	8.4 ^a^ ± 0.3	8.3 ^a^ ± 0.2	8.1 ^a^ ± 0.3	8.1 ^a^ ± 0.3
HDM-LH	8.7 ^a^ ± 0.4	8.5 ^a^ ± 0.3	8.2 ^a^ ± 0.2	8.2 ^a^ ± 0.3	8.1 ^a^ ± 0.3

Explanatory notes: See Table 3. Values within the same column with different superscript letters (a–d) are significantly different at *p* < 0.05.

**Table 5:** The sensory evaluation of full-fat cheese without whey separation during refrigerated storage for 28 days (mean and SD).

Sensory Parameter	Appearance	Consistency	Taste	Smell	Overall Acceptability
Sample code	1st week				
LDM	4.80 ^a^ ± 0.20	4.78 ^a^ ± 0.22	4.50 ^a^ ± 0.30	4.67 ^b^ ± 0.20	4.82 ^a^ ± 0.20
LDM-LH	4.82 ^a^ ± 0.16	4.77 ^a^ ± 0.21	4.45 ^a^ ± 0.23	4.53 ^b^ ± 0.11	4.83 ^a^ ± 0.18
HDM	4.87 ^a^ ± 0.11	4.81 ^a^ ± 0.20	4.67 ^a^ ± 0.20	4.97 ^a^ ± 0.01	5.00 ^a^ ± 0.03
HDM-LH	4.88 ^a^ ± 0.13	4.83 ^a^ ± 0.11	4.76 ^a^ ± 0.21	4.98 ^a^ ± 0.01	5.00 ^a^ ± 0.01
	4th week				
LDM	4.78 ^a^ ± 0.09	4.68 ^a^ ± 0.12	4.49 ^b^ ± 0.11	4.63 ^b^ ± 0.18	4.79 ^a^ ± 0.13
LDM-LH	4.77 ^a^ ± 0.08	4.69 ^a^ ± 0.08	4.48 ^a,b^ ± 0.21	4.55 ^b^ ± 0.11	4.80 ^a^ ± 0.20
HDM	4.80 ^a^ ± 0.11	4.70 ^a^ ± 0.30	4.67 ^a^ ± 0.08	4.91 ^a^ ± 0.04	4.97 ^a^ ± 0.05
HDM-LH	4.85 ^a^ ± 0.07	4.69 ^a^ ± 0.20	4.77 ^a^ ± 0.10	4.99 ^a^ ± 0.10	4.98 ^a^ ± 0.05

Explanatory notes: See Table 3. Values within the same column with different superscript letters are significantly different at *p* < 0.05.

## Data Availability

The original contributions presented in this study are included in the article. Further inquiries can be directed to the corresponding author.
